# Evaluating the accuracy of point of care testing compared to standard laboratory testing among inborn infants in the neonatal intensive care unit

**DOI:** 10.21203/rs.3.rs-7382648/v1

**Published:** 2025-09-01

**Authors:** Lindsay Holzapfel, Isha Parikh, Sepideh Saroukhani, Matthew Rysavy, Mar Romero-Lopez, Brian Chang

**Affiliations:** University of Texas, Houston Health Science Center; University of Texas, Houston Health Science Center; University of Texas, Houston Health Science Center; University of Texas Health Science Center at Houston; University of Texas, Houston Health Science Center; University of Texas, Houston Health Science Center

## Abstract

**Objective:**

Point-of-care (POC) testing offers expedited results with lower blood volume requirements than central laboratory (CL) tests, particularly beneficial for low-birth-weight infants.

**Methods:**

A retrospective cohort of 118 patients with paired POC and CL tests was performed within one hour during the first 14 postnatal days. Differences and agreement were assessed using paired t-tests and Lin’s concordance correlation coefficient (CCC).

**Results:**

Differences were observed between POC and CL measurements: sodium (6.0 mEq/L, CCC = 0.57), potassium (0.1 mEq/dL, CCC = 0.82), chloride (4.4 mEq/L, CCC = 0.70), glucose (3.5 mg/dL, CCC = 0.97), hemoglobin (−0.04 g/dL, CCC = 0.98) and hematocrit (−0.6%, CCC = 0.97). Differences were consistent across lab results, gestational ages, birthweights, and clinical factors.

**Conclusions:**

POC results differed from CL results in sodium and chloride, with little difference in potassium, glucose, hemoglobin, and hematocrit. POC testing may reduce blood volume and provide rapid results for decision-making.

## INTRODUCTION

Point-of-care (**POC)** testing offers expedited results for timely intervention in the neonatal intensive care unit (**NICU**). POC testing may also be advantageous for infants with very low birth weights because it requires a lower blood volume. POC testing for glucose is often used at the bedside for infants and adults, and its comparable performance to gold-standard central laboratory (**CL**) testing is well established (1). Other key analytes relevant in critically ill patients, such as lactate, INR, C-reactive protein, and procalcitonin, are validated using POC assays in infant populations (2–4). Few studies demonstrate the accuracy of sodium, bilirubin, chloride, and potassium with POC testing among infants (5, 6). Still, there is a paucity of evidence available across a broad spectrum of gestational age, birth weight, and illness severity, leaving gaps in our understanding of POC testing accuracy for infants of all ages and birth weights.

POC testing has the potential to have significant clinical benefits for preterm infants. Notable differences between POC and CL testing include the blood volume needed for various laboratory tests and agreement for each measurement among all ages and weights. This issue is particularly concerning for the smallest and youngest infants, as their total blood volume is significantly lower than that of term infants or older children. For a 500 g infant, with an estimated total blood volume of 90 mL/kg, blood volume is estimated as ~ 45 ml. When frequent laboratory testing is required—often several times daily—cumulative blood loss can quickly become substantial. CL testing generally requires blood volumes several times that of POC tests for similar measures (7).

Point-of-care testing may expedite diagnosis and treatment in NICUs. However, understanding the potential for differences in results is key for optimal clinical interpretation and management, mainly when most electronic medical records include laboratory values obtained from multiple sources. The primary aim of this study was to compare the accuracy of POC testing using the GEM Premier 5 000 blood gas analyzer (Werfen, Barcelona) deployed in the POC setting (8) against hospital CL testing for sodium, potassium, chloride, glucose, hemoglobin, and hematocrit at our hospital. The secondary aim was to measure the differences across the range of laboratory values, gestational age, birth weight, infant severity of illness, weight loss, and fluid intake.

## METHODS

This retrospective cohort study included inborn patients admitted to the Children’s Memorial Hermann Hospital NICU (Houston, Texas) from September 2022 through May 2024 who received POC testing. Inborn infants were included if they received both POC and CL testing, drawn within one hour of each other during the first 14 postnatal days. At our center, infants receive frequent, daily laboratory testing in the first 14 postnatal days, and this period was selected to produce the most matched POC and CL samples. We identified approximately 1 000 inborn infants from the selected dates who received POC testing during hospitalization. Each eligible infant was assigned a unique numeric identifier. To obtain a representative subset for manual chart review, we selected a simple random sample of 118 infants (approximately 10–15% of the eligible population) using an online random number generator (14). No samples were excluded.

The GEM Premier 5 000 blood gas analyzer (8) can test arterial, venous, capillary, and mixed venous samples with as little as 0.2 mL (or less if capillary) of blood volume. Sodium, potassium, and chloride testing on the GEM employs direct potentiometry; glucose testing involves amperometric measurement of the oxidation of hydrogen peroxide; bilirubin and total hemoglobin testing are based on optical absorbance; and hematocrit is measured by electrical conductivity. In contrast, CL electrolyte measurement is via indirect potentiometry; glucose, bilirubin, and hemoglobin are done by optical absorbance; and hematocrit is determined using hydrodynamically focused DC detection. POC results can be available in about 2 minutes. In contrast, CL turnaround time often requires up to 30–45 minutes, the bulk of which is due to specimen transit time, sample accessioning, and centrifugation.

Infant demographics were recorded. A Score for Neonatal Acute Physiology and SNAP Perinatal Extension (**SNAPPE-II**) for each infant was recorded to quantify infant severity of illness and was calculated for the first 12 hours after birth using the online calculator (9). SNAPPE-II values range from 0 to 162, with an increased SNAPPE-II score indicating a higher risk for infant mortality. Ethical approval for waiver of consent for this study was obtained from the University of Texas Houston Health Science Center Institutional Review Board. All de-identified data were securely stored in a RedCap database, and patient confidentiality was strictly maintained.

For statistical analysis, POC testing and gold-standard CL testing for electrolytes were compared using a paired t-test or Wilcoxon signed-rank test as its non-parametric equivalent where appropriate. The mean difference and 95% confidence interval with a limit of agreement between POC and CL was reported for each electrolyte. In addition, Lin’s Concordance Correlation Coefficient (CCC) was calculated to quantify the agreement between the POC and CL paired values. Bland–Altman analyses were performed to quantify and visualize the magnitude of agreement and bias. Variability of the results for each of the lab measurements by potential modifiers, including extremely low birth weight (< 1 000 g), extremely low gestational ages (< 25 weeks, 25 1/7 to 29 6/7 weeks, > 30 weeks), SNAPPE-II scores (< 32), and percentage of weight loss from birth (< 15%) were explored by including the interaction term in linear mixed model with subject ID for paired values as random effect. Daily weights were missing for 124 of the daily measurement, the percent weight loss was only calculated on days where both weights were available. All statistical tests will be conducted at 0.05 level of significance using SAS 9.4 statistical software.

## RESULTS

This study included 118 inborn NICU patients with birth weights of 2 115 ± 1 043 g (mean ± SD) and gestational ages of 33.1 ± 5.3 weeks (Table 1). The median SNAPPE-II score for the total sample was 32. Of the recorded POC samples, 69.7% were arterial blood samples, with 23.4% capillary and 6.9% venous blood samples.

There were 609 paired samples for sodium, 657 pairs for potassium, 596 pairs for chloride, 593 pairs for glucose, 325 pairs for hemoglobin, and 428 pairs for hematocrit. Table 2 reports the mean differences between CL and POC testing. Comparing CL to POC results, the mean difference was 6.0 mEq/L for sodium (95% CI 5.8, 6.2, p <0.001), 0.1 mEq/L for potassium (95% CI 0.1, 0.2, p <0.001), 4.4 mEq/L for chloride (95% CI 4.2, 4.7, p <0.001), 3.5 mg/dL for glucose (95% CI 2.5, 4.4 CI, p <0.001), 0.04 g/dL for hemoglobin (95% CI −0.1, 0.01, p=0.08), and 0.6 for hematocrit (95% CI −0.8, −0.4, p<0.001). For each lab test, the upper and lower limits of agreement (mean difference ± 1.96 SD) are shown in Table 2, representing the 95% range of differences between CL and POC testing. Sodium had the lowest CCC, 0.57, whereas glucose, hemoglobin, and hematocrit had high concordance (0.95–0.99).

**Figure 1:**
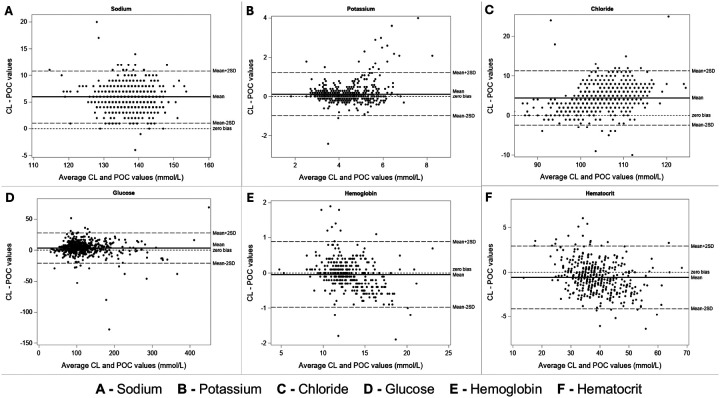
Bland Altman Plots of Central Laboratory vs Point-of-Care. **Panel A:** Bland Altman plot of Sodium CL vs POC values. Dashed lines show 95% limits of agreement, and the solid line indicates the mean difference between the CL and POC. Positive mean difference (bias) of 6 between CL and POC. 95% Confidence interval (CI) Limits of agreement are between 1.2 and 10.7. **Panel B:** Bland Altman plot of Potassium CL vs POC values. There is a slightly positive mean difference (bias) of 0.1 between CL and POC. 95% Limits of agreement are between −0.96 and 1.2. Some variation of the results above the potassium value of 6. **Panel C:** Bland Altman plot of Chloride CL vs POC values. Positive mean difference (bias) of 4.4 between CL and POC. 95% Limits of agreement are between −2.2 and 11.1. **Panel D:** Bland Altman plot of Glucose CL vs POC values. Positive mean difference (bias) of 3.4 between CL and POC. 95% Limits of agreement are between −20.2 and 27.2. Some variation of results above the glucose value of 200. **Panel E:** Bland Altman plot of Hemoglobin CL vs POC values. Negative mean difference (bias) of −0.05 between CL and POC. 95% Limits of agreement are between −1.0 and 0.9. **Panel F:** Bland Altman plot of Hematocrit CL vs POC values. Negative mean difference (bias) of −0.6 between CL and POC. 95% Limits of agreement are between −4.1 and 2.9.

Bland-Altman plots show variation in the difference between POC and CL values across the range of results (Figure 1). Differences between CL and POC were consistent across the range of results except for potassium and glucose, which had increased variability at extreme values.

**Figure 2:**
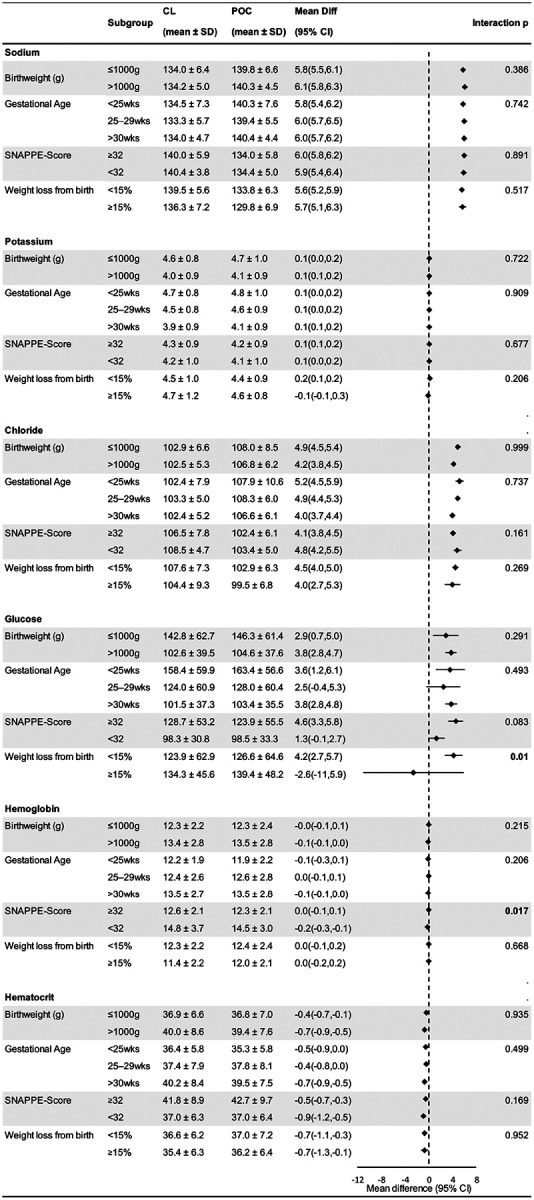
Comparison of POC and CL values by potential modifiers Comparison of paired POC and CL measurements for sodium, potassium, chloride, glucose, hemoglobin, and hematocrit, stratified by birthweight, gestational age, SNAPPE-II score, and weight loss from birth. The table presents mean ± SD, mean difference (95% CI), and interaction p-values. The accompanying forest plots display mean differences with 95% Cis. The vertical dashed line represents no difference (total agreement) between POC and CL. Filled squares indicate mean differences, with bars showing precision. Points to the right indicate POC overestimation and to the left indicate underestimation. CIs crossing the dashed line indicate no statistically significant difference.

A forest plot (Figure 2) was created to visualize the mean differences between POC and CL measurements across clinical subgroups. In Figure 2, there was no effect modification of any of the following factors on differences between POC and CL results for sodium, potassium, chloride, and hematocrit values: birthweight (≤1 000 or > 1 000 g), gestational age (<25 weeks, 25 1/7 to 29 6/7 weeks, ≥30 weeks), SNAPPE-II score (<32 or ≥32 median sample score), weight loss from birth (<15% or ≥15%). The agreement between glucose measured at CL and POC differed by percent weight loss from birth (interaction p = 0.01). Specifically, for infants with weight loss <15%, the POC value was significantly higher than CL value [Mean difference 4.2, 95% CI (2.7, 5.7)], whereas among infants with a percent weight loss ≥15% from birth the difference was in opposite direction and not significant [Mean difference −2.6, 95% CI (−11, 5.9)]. Agreement between CL and POC hemoglobin values differed by SNAPPE-II score (interaction p = 0.017) with a mean difference (95% CI) of 0 (−0.1,0.1) for SNAPPE-II scores at ≥32 and a mean difference (95% CI) of −0.2 (−0.3, −0.1) for SNAPPE-II scores <32.

## DISCUSSION

The primary aim of this study was to compare POC testing using the GEM Premier 5 000 blood gas analyzer set up in a POC environment (8) to CL testing for sodium, potassium, chloride, glucose, hemoglobin, and hematocrit at our hospital. POC results differed from CL results in sodium and chloride measures, with minimal difference observed in potassium, hemoglobin, and hematocrit measures. These differences remained consistent across the range of lab results and birth weights, gestational ages, and SNAPPE-II scores. While most hypothesized potential effect modifiers showed no significant interactions, possible interactions were observed for glucose and percent weight loss from birth and hemoglobin and SNAPPE-II score. However, given the relatively small sample size in this subgroup, this relationship should be interpreted with caution clinically. Assessing possible variations of result by modifiers were exploratory in this study and require replication in larger studies

POC compared to CL testing in this single-center retrospective cohort study showed a consistent mean negative bias in sodium measurement of 6 mEq/L across a wide range of sodium values (120 mEq/L – 150 mEq/L), which may affect clinical decision-making when values approach thresholds for hyper- and hyponatremia. Sodium measures with moderate agreement and variability. Outside the normal range for potassium (3.5–5.0 mEq/L) and for elevated glucose (> 180 mg/dL), POC and CL testing had greater variability. Clinically, this may be explained by the general knowledge that hemolysis may produce erroneously high potassium. As a standard practice at our institution, when an elevated potassium value (> 6.5 mEq/L) is obtained by one measure, a confirmatory lab may be ordered, likely explaining the trend that we observe of greater variability at the extremes of the results range. Variability between POC and CL testing for glucose may be partly explained by the established 5–7% per hour glucose concentration loss from blood samples’ glycolysis in CL testing, which can be accelerated in higher temperatures and longer wait times from collection to analysis (11). POC and CL results were similar across their ranges for other lab measures.

Cost analysis was beyond the scope of this study, but the potential for blood volume savings (0.2 mL vs 1 mL per test) and faster turnaround time (2 minutes vs 30–45 minutes), as well as differences in financial cost to patients or the healthcare system, are relevant considerations in decision making about use of POC vs CL tests. POC testing may be more expensive on a per-test basis due to the limited capacity of cartridges and the greater hands-on time required to run each sample. However, the convenience and faster turnaround can offer meaningful clinical benefits that may justify the higher cost in specific settings.

This study is limited by the nature of its retrospective design and small sample size, with infants randomly selected from all inborn NICU admissions during the study period. However, to our knowledge, it is the largest study to date on this topic. Increasing survival among the smallest and most premature infants, comprising 25% of our cohort, may make POC testing particularly important to limit blood volumes. Because of the retrospective nature of this study, we were unable to determine the rationale for repeated samples such as potassium or glucose, nor were we able to assess factors relevant to result reporting, such as hemolysis or sample origin (venous, arterial, or capillary). However, most samples were likely duplicated due to local clinical practice habits that include obtaining a POC blood gas and CL electrolyte measurement daily in extremely preterm infants during the first 2 postnatal weeks.

## CONCLUSION

In this single-center retrospective study of extremely preterm infants during the first 2 postnatal weeks, we observed minimal differences in POC measures of potassium, hemoglobin, and hematocrit compared to CL measures. POC results differed more substantially from CL results in sodium and chloride measures. POC testing is generally comparable to CL testing in routine and critical care environments, with the advantage of reducing blood sample volumes while providing far more rapid results for clinical decision-making. Multicenter validation studies are necessary to enhance the generalizability of these findings.

## Figures and Tables

**Table 1:** Baseline infant characteristics.

Characteristic	n = 118
Birth weight, *g*^1^	2115 ± 1 043
<500 g^2^	1 (0.8)
501–1 000 g^2^	29 (24.6)
1 001–1 500 g^2^	11 (9.3)
>1 500 g^2^	77 (65.3)
Gestational age, *weeks*^1^	33.1 ± 5.3
<25^2^	10 (8.5)
25 1/7 to 29 6/7^2^	29 (24.6)
>30^2^	79 (66.9)
Male sex^2^	72 (61)
Race^2^	
White	33 (28)
Black	35 (29.7)
Asian	10 (8.5)
Unknown	40 (33.9)
C-section delivery^2^	86 (74.8)
SNAPPE-II score^3^	32 (16, 49)
Blood compartment for sample^2^	
Arterial	545 (69.7)
Venous	54 (6.9)
Capillary	183 (23.4)

1 Mean ± SD;

2 n(%);

3 Median (IQR). SNAPPE-II - Score for Neonatal Acute Physiology and SNAP Perinatal Extension(9).

**Table 2:** Comparison of CL and POC values.

	n (pairs)	CL	POC	Mean Difference (95% CI)	Upper limit of agreement (Mean + 1.96 SD)	Lower limit of agreement (Mean − 1.96 SD)	CCC	p-value
Sodium (mEq/L)	609	140.2 ± 5.3	134.2 ± 5.5	6.0 (5.8, 6.2)	10.7 (10.4, 11.1)	1.2 (0.8, 1.5)	0.57	<0.001
Potassium (mEq/L)	657	4.3 ± 1.0	4.2 ± 0.9	0.1 (0.1, 0.2)	1.2 (1.1, 1.3)	−1.0 (−1.0, −0.9)	0.82	<0.001
Chloride (mEq/L)	596	107.2 ± 7.1	102.6 ± 5.8	4.4 (4.2, 4.7)	11.1 (10.6, 11.6)	−2.2 (−2.7, −1.8)	0.7	<0.001
Glucose (mg/dL)	593	118.4 ± 50.7	117.0 ± 52.7	3.5 (2.5, 4.4)	27.2 (25.5, 28.8)	−20.2 (−21.9, −18.6)	0.97	<0.001
Hemoglobin (g/dL)	325	13.2 ± 2.8	13.0 ± 2.6	−0.04 (−0.1, 0.01)	0.9 (0.8, 1.0)	−1.0 (−1.0, −0.9)	0.98	0.08
Hematocrit	428	38.6 ± 7.5	38.8 ± 8.0	−0.6 (−0.8, −0.4)	2.9 (2.6, 3.1)	−4.1 (−4.3, −3.8)	0.97	<0.001

Paired t-test, mean ± SD; CCC: Lin’s concordance correlation coefficient (CCC); CI: confidence interval; CL: central lab; POC: point of care.

## Data Availability

The datasets generated and analyzed during the current study are available from the corresponding author upon request.
